# DeepImpute: an accurate, fast, and scalable deep neural network method to impute single-cell RNA-seq data

**DOI:** 10.1186/s13059-019-1837-6

**Published:** 2019-10-18

**Authors:** Cédric Arisdakessian, Olivier Poirion, Breck Yunits, Xun Zhu, Lana X. Garmire

**Affiliations:** 1 Department of Information and Computer Science, University of Hawaii at Manoa, Honolulu, HI 96816 USA; 2 Department of Epidemiology, University of Hawaii Cancer Center, 701 Ilalo Street, Honolulu, HI 96813 USA; 3 Department of Molecular Biology and Bioengineering, University of Hawaii at Manoa, Honolulu, HI 96816 USA; 4 Department of Computational Medicine and Bioinformatics, University of Michigan, Ann Arbor, MI 48105 USA

**Keywords:** RNA-seq, Single-cell, Imputation, Deep learning, Machine learning, Neural network, Dropout, DeepImpute

## Abstract

Single-cell RNA sequencing (scRNA-seq) offers new opportunities to study gene expression of tens of thousands of single cells simultaneously. We present DeepImpute, a deep neural network-based imputation algorithm that uses dropout layers and loss functions to learn patterns in the data, allowing for accurate imputation. Overall, DeepImpute yields better accuracy than other six publicly available scRNA-seq imputation methods on experimental data, as measured by the mean squared error or Pearson’s correlation coefficient. DeepImpute is an accurate, fast, and scalable imputation tool that is suited to handle the ever-increasing volume of scRNA-seq data, and is freely available at https://github.com/lanagarmire/DeepImpute.

## Introduction

The RNA sequencing technologies keep evolving and offering new insights to understand biological systems. In particular, single-cell RNA sequencing (scRNA-seq) represents a major breakthrough in this field. It brings a new dimension to RNA-seq studies by zooming in to the single-cell level. Currently, various scRNA-seq platforms are available such as Fluidigm- and Drop-Seq-based methods. While Drop-Seq can process thousands of cells in a single run, Fluidigm generally processes fewer cells but with a higher coverage. In particular, 10X Genomics’ platform is gaining popularity in the scRNA-seq community due to its high yield and low cost per cell. Consequently, an increasing amount of studies have taken advantage of these technologies to discover new cell types [1, 2], new markers for specific cell types [1, 3, 4], and cellular heterogeneity [4–9].

Despite these advantages, scRNA-seq data are very noisy and incomplete [10–12] due to the low starting amount of mRNA copies per cell. Datasets with more than 70% missing (zero) values are frequently observed in an scRNA-seq experiment. These apparent zero values could be truly zeros or false negatives. The latter phenomenon is called “dropout” [13] and is due to failure of amplification of the original RNA transcripts. Among genes of various lengths, shorter genes were more likely to be dropped out [14]. Such bias may increase further during the subsequent amplification steps. As a result, dropout can affect downstream bioinformatics analysis significantly, such as clustering [15] and pseudo-time reconstruction [16], as it decreases the power of the studies and introduces biases in gene expression. To correct such issue, analysis platforms such as Granatum [17] have included an imputation step, in order to improve the downstream analysis.

Currently, several imputation algorithms have been proposed, based on different principles and models. MAGIC [18] focuses on cell/cell interactions to build a Markov transition matrix and smooth the data. ScImpute [19] builds a LASSO regression model for each cell and imputes them iteratively. SAVER [20] is a Bayesian-based model using various prior probability functions. DrImpute [21] is a clustering-based method and uses a consensus strategy: it estimates a value with several cluster priors or distance matrices and then imputes by aggregation. VIPER is a recent published statistical method that looks at cell/cell interaction to fit a linear model for each cell. Instead of using a LASSO regression as for scImpute, the authors use a hard thresholding approach to limit the number of predictors [22]. Most recently, DCA builds an auto-encoder to model the genes distribution using a zero inflated negative binomial prior. To this end, the auto-encoder tries to predict the genes’ mean, standard deviation, and dropout probability [23]. As the low quality of the scRNA-seq datasets continues to be a bottleneck while the measurable cell counts keep increasing, the demand for faster and scalable imputation methods also keeps increasing [23–25]. While some of these earlier algorithms may improve the quality of original datasets and preserve the underlying biological variance [26], most of these methods demand extensive running time, impeding their adoption in the ever-increasing scRNA-seq data space.

Here, we present a novel algorithm, DeepImpute, as the next generation imputation method for scRNA-seq data. DeepImpute is short for “Deep neural network Imputation”. As reflected by the name, it belongs to the class of deep neural-network models [27–29]. Recent years, deep neural network algorithms have gained much interest in the biomedical field [30], ranging from applications from extracting stable gene expression signatures in large sets of public data [31] to stratify phenotypes [32] or impute missing values [33] using electronic health record (EHR) data. In this report, we construct DeepImpute models by splitting the genes into subsets and build sub-networks to increase its efficacy and efficiency. Using accuracy metrics, we demonstrate that DeepImpute performs better than the six other recently published imputation methods mentioned above (MAGIC, DrImpute, ScImpute, SAVER, VIPER, and DCA). It also improved the downstream analysis results, on clustering using both real and simulated datasets, as well as on differential expression using a simulated dataset. We additionally show the superiority of DeepImpute over the other methods in terms of computational running time and memory use. Moreover, DeepImpute allows to train the model with a subset of data to save computing time, with little sacrifice on the prediction accuracy. In summary, DeepImpute is a fast, scalable, and accurate next generation imputation method capable of handling the ever-increasing scRNA-seq data.

## Results

### Overview of the DeepImpute algorithm

**Fig. 1:**
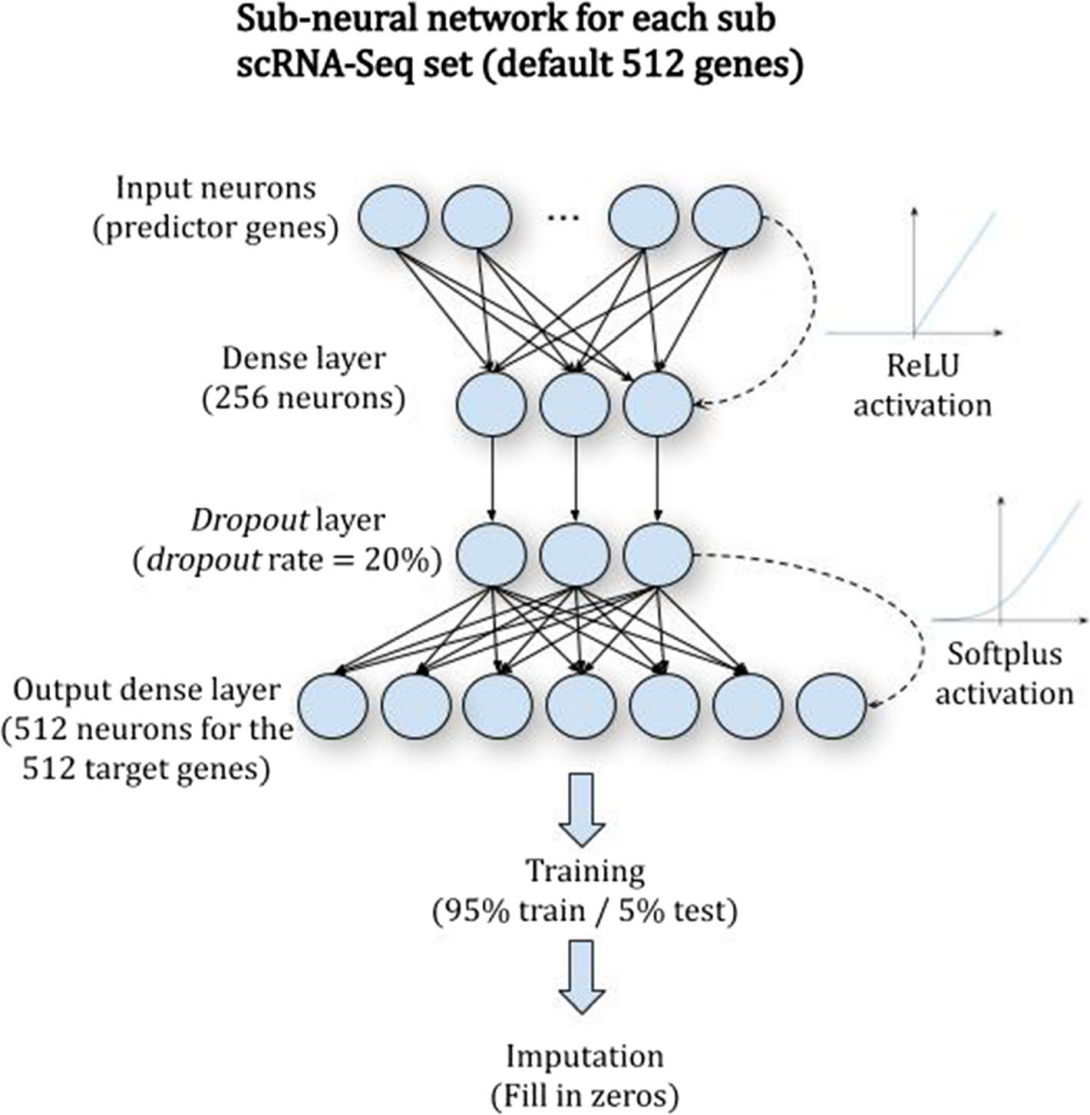
(Sub) Neural network architecture of DeepImpute. Each sub-neural network is composed of four layers. The input layer is genes that are highly correlated with the target genes in the output layer. It is followed by a dense hidden layer of 256 neurons dense layer and a *dropout* layer (*dropout* rate = 20%). The output layer consists of a subset of target genes (default *N* = 512), whose zero values are to be imputed

DeepImpute is a deep neural network model that imputes genes in a divide-and-conquer approach, by constructing multiple sub-neural networks (Additional file 1: Figure S1). Doing so offers the advantage of reducing the complexity by learning smaller problems and fine-tuning the sub-neural networks [34]. For each dataset, we select to impute a list of genes, which have a certain variance over mean ratio (default = 0.5). Each sub-neural network aims to understand the relationship between the input genes (input layer) and a subset of target genes (output layer) (Fig. 1). Users can set the size of the target genes, and we set 512 as the default value, as it offers a good trade-off between speed and stability. As shown in Fig. 1, each sub-neural network is composed of four layers. The input layer consists of genes that are highly correlated with the target genes, followed by a 256-neuron dense hidden layer, a *dropout* layer with 20% *dropout* rate (note: not the dropout rate in the single cell data matrix) of neurons which avoid overfitting (Additional file 2: Figure S1), and the output neurons made of the abovementioned target genes. We use rectified linear unit (ReLU) as the default activation function and train each sub-model in parallel by splitting the data to train (95% of the cells) and test (5%) data. We stop the training if the test loss does not improve for 5 consecutive epochs or the number of epochs exceeds 500, whichever is smaller. Because of the simplicity of each sub-network, we observe very low variability due to hyperparameter tuning. As a result, we set the default parameters for batch size at 64 and learning rate at 0.0001. Further information about the network parameters are described in the “Methods” section. In the following sections, we describe the comprehensive evaluations of DeepImpute.

### DeepImpute is the most accurate among imputation methods on scRNA-seq data

We tested the accuracy of imputation on four publicly available scRNA-seq datasets (Additional file 3: Table S1): two cell lines, Jurkat and 293T (10X Genomic); one mouse neuron cells dataset (10X Genomics); and one mouse interfollicular epidermis dataset deposited in GSE67602. We compared DeepImpute with six other state-of-the-art, representative algorithms: MAGIC, DrImpute, ScImpute, SAVER, VIPER, and DCA. Since the real dropout values are unknown, we evaluated the different methods by randomly masking (replacing with zeros) a part of the expression matrix of a scRNA-seq dataset (Additional file 1: Figure S2) and then measure the differences between the inferred and actual values of the masked data. In order to mimic a more realistic dropout distribution, we estimated the masking probability function from the data (see the “Methods” section). We measured the accuracies using the two metrics on the masked values: Pearson’s correlation coefficient and mean squared error (MSE), as done earlier [20, 35].

**Fig. 2:**
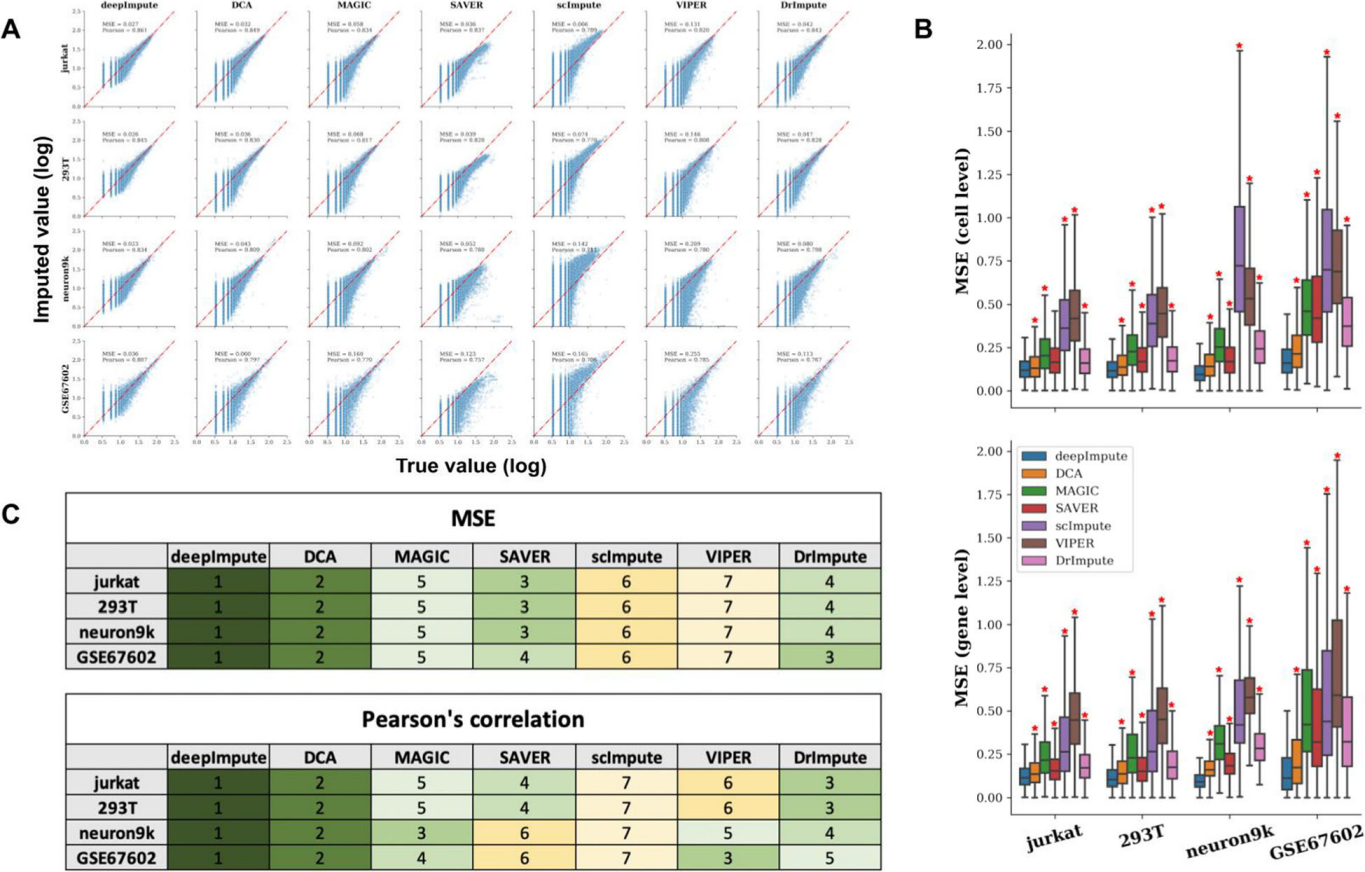
Accuracy comparison between DeepImpute and other competing methods. **a** Scatter plots of imputed vs. original data masked. The *x*-axis corresponds to the true values of the masked data points, and the *y*-axis represents the imputed values. Each row is a different dataset, and each column is a different imputation method. The mean squared error (MSE) and Pearson’s correlation coefficients (Pearson) are shown above each dataset and method. The rankings of these methods are shown below the figure in color coding. **b** Bar graphs of cell-cell and gene-gene level MSEs between the true (masked) and imputed values, based on those in **a**. Asterisk indicates statistically significant difference (*P* < 0.05) between DeepImpute and the imputation method of interest using the Wilcoxon rank-sum test. Color labels for all imputation methods are shown in the figure (**c**). Ranking of each method for all four datasets for both overall MSE and Pearson's correlation coefficient

Figure 2 shows all the results of imputation accuracy metrics on the masked data. DeepImpute successfully recovers dropout values from all ranges, introduces the least distortions and biases to the masked values, and yields both the highest Pearson’s correlation coefficient and the best (lowest) MSE in all datasets (Fig. 2a and c). DCA, another neuron-network-based method, has the second best performance after DeepImpute, based on both MSE and Pearson’s correlation coefficient. On the contrary, other methods present various issues: VIPER tends to underestimate the original values, as reflected by the largest MSEs. scImpute has the widest range of variations among imputed data and generates the lowest Pearson’s correlations. MAGIC, SAVER, and DrImpute have intermediate performances compared to other methods. However, SAVER persistently underestimate the values, especially among the highly expressed genes. We further examined MSE distributions calculated on the gene and cell levels (Fig. 2b). DeepImpute is the clear winner with consistently the best (lowest) MSEs both at gene and cell levels on all datasets, which are significantly lower than all other imputation methods (*p* < 0.05). scImpute and VIPER give the two highest MSEs at the cell level, whereas VIPER consistently has the highest MSE at the gene level (Fig. 2b). Other methods are ranked in between, with varying rankings depending on the datasets and gene or cell level. As internal controls, we also compared DeepImpute (with ReLU activation) with 2 variant architectures: the first one with no hidden layers and the second one with the same hidden layers but using linear activation function (instead of ReLU). As shown in Additional file 2: Figure S2, DeepImpute (with ReLU activation) yields Pearson’s correlation coefficients and MSEs that are either on par or better than the two other variant architectures. For GSE67602 and neuron9k datasets generated from complex animal primary tissues, DeepImpute (with ReLU activation) performs better; for Jurkat and 293T datasets generated from cell lines, the results are comparable. This suggests that DeepImpute (with ReLU activation) handles complex datasets better than its variants. In summary, DeepImpute yields the highest accuracy in the datasets studied, among the imputation methods in comparison.

### DeepImpute improves the gene distribution similarity with FISH experimental data

**Fig. 3:**
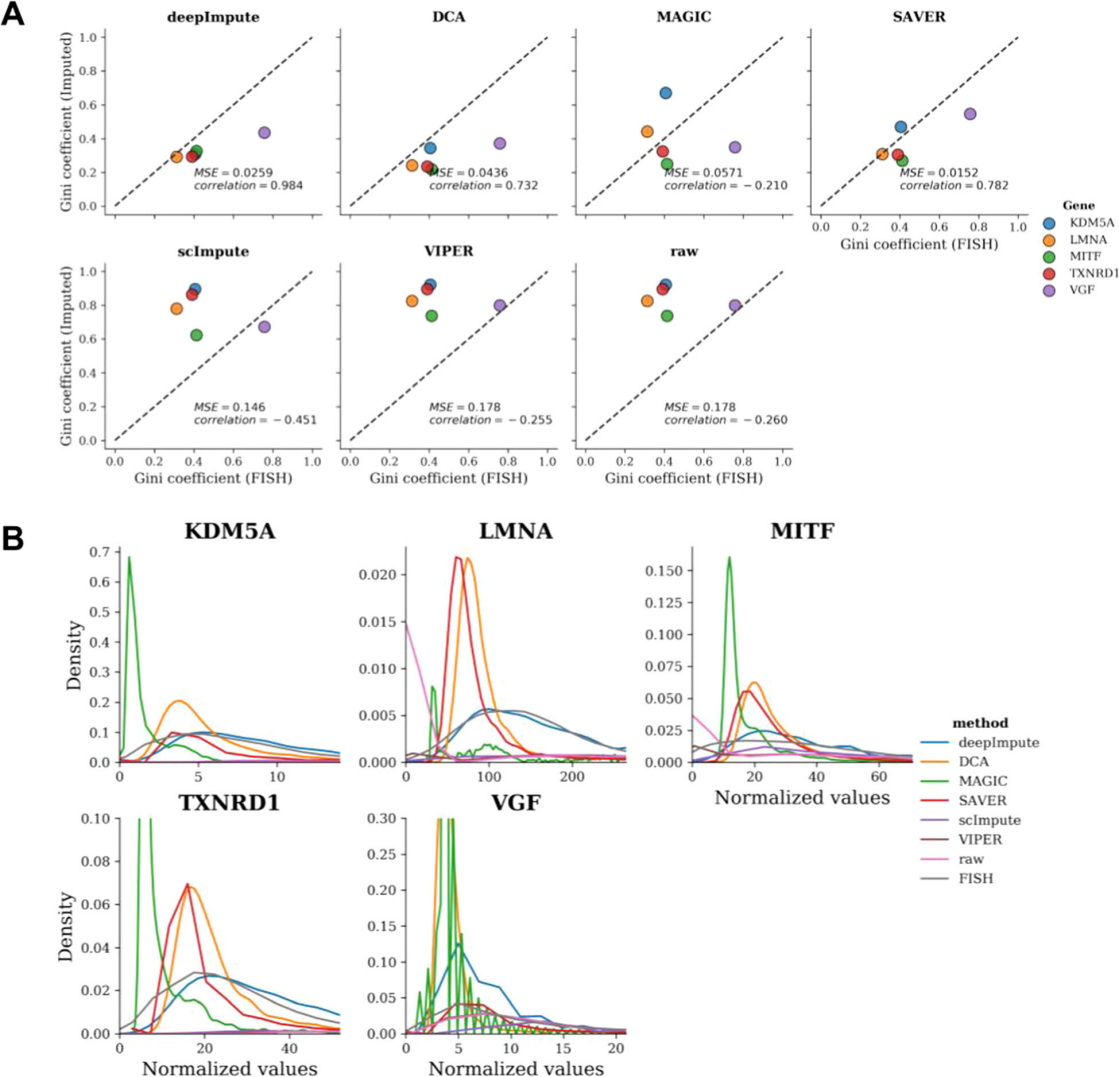
Comparison among imputation methods using RNA FISH data. **a** Scatter plots of GINI coefficients from the imputed (or raw) vs. FISH data. The *x*-axis is the “true” GINI coefficient as determined by FISH experiments, and the *y*-axis is the imputed (or raw) GINI coefficient. The Pearson’s correlation coefficients (Pearson) and mean squared error (MSE) are shown for each method. Colors represent different genes: KDM5A (blue), LMNA (yellow), MITF (Green), TXNRD1 (red), and VGF (brown). **b** Gene distributions for seven imputation methods: DeepImpute (blue), DCA (yellow), MAGIC (green), SAVER (red), scImpute (purple), VIPER (brown), raw (pink), and FISH (gray) data

Another way to assess the imputation efficiency is through experimental validation on scRNA-Seq data. Single-cell RNA FISH is such a method that directly detects a small number of RNA transcripts in a single cell. Torre et al. measured the gene expression of a melanoma cell line using both RNA FISH and Drop-Seq and compared their distribution using their GINI coefficients (see the “Methods” section) [36]. Similarly, we compared the same list of genes using their GINI coefficients of RNA FISH vs. those after imputation (or raw scRNA-seq data). DrImpute could not handle the large cell size and was omitted from comparison. Comparing to Pearson’s correlation coefficient between RNA FISH and the raw scRNA-seq data (− 0.260), three methods, DeepImpute, SAVER, and DCA, have the top 3 most improved and positive correlation coefficients, with values of 0.984, 0.782, and 0.732, respectively. VIPER barely changed the GINI coefficients, whereas scImpute had a correlation coefficient (− 0.451) even lower than the raw scRNA-seq dataset (Fig. 3a). For MSE, all other imputation methods achieved better (smaller) MSEs compared to the raw scRNA-seq results (MSE = 0.281), except VIPER which gives the same MSE as raw data. Echoing the results of correlation coefficient, three methods, SAVER, DeepImpute, and DCA, give the lowest MSEs. DeepImpute is the second most accurate method with an MSE (MSE = 0.0256), closely after SAVER (MSE = 0.0152) and followed by DCA (MSE = 0.0436). Additionally, we compared the distributions of each gene before and after various imputation methods, as well as in FISH experiments (Fig. 3b). Overall, DeepImpute (blue curves) yields the most similar distributions to those of FISH experiments (gray curves) for three of five genes (LMNA, MITF, and TXNRD1), with K-S test statistics of 0.08, 0.15, and 0.18, respectively. For KDM5A, it achieved 2nd best K-S statistics 0.18, almost the same as DCA (0.17). It does not perform as well for gene VGF (K-S statistic of 0.44), which has over 40% zero values even in RNA-FISH data (56% in raw Drop-Seq data). Altogether, the FISH validation results clearly show that DeepImpute improves the data quality by imputation.

### DeepImpute improves downstream functional analysis

**Fig. 4:**
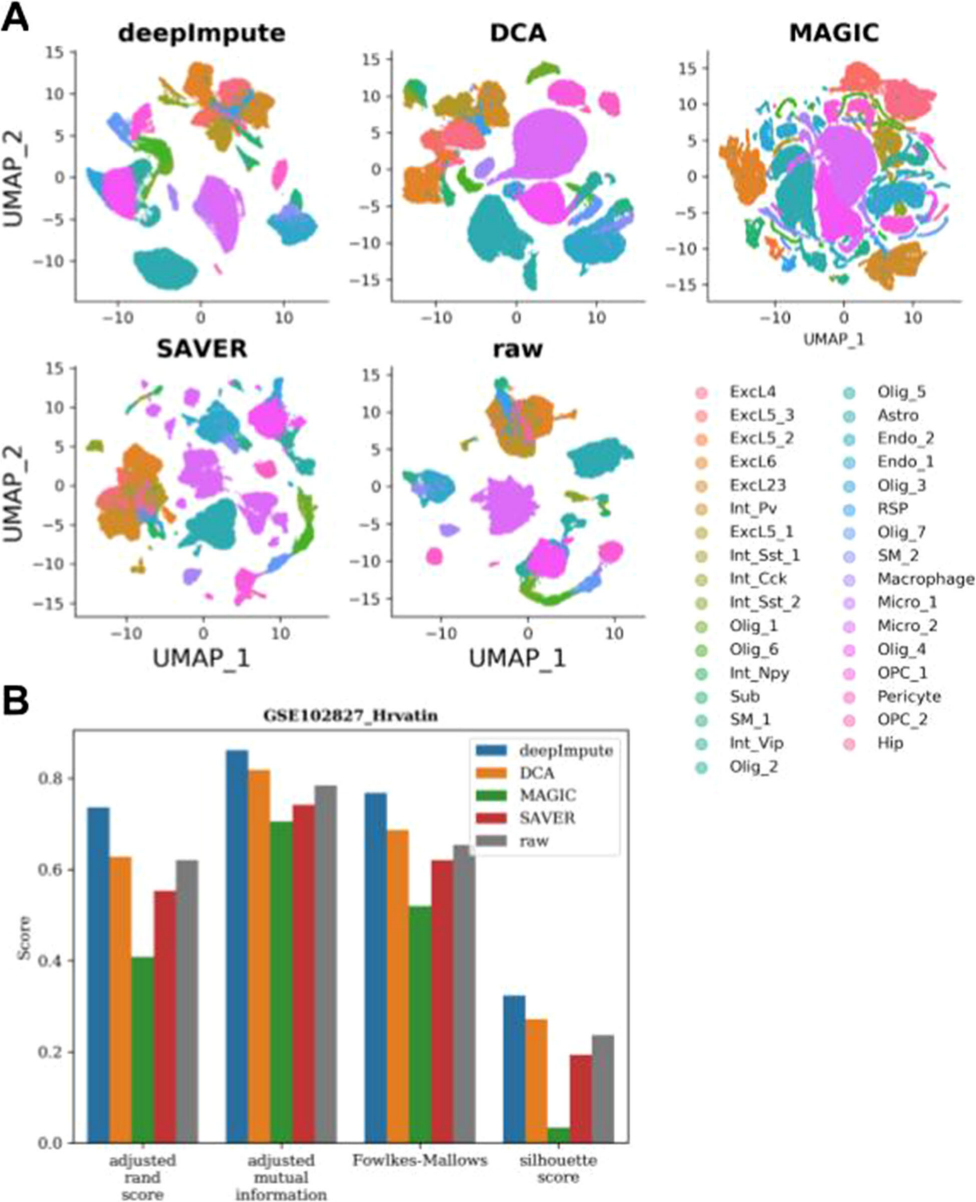
Comparison on effect of imputation on downstream function analysis of the experimental data (GSE102827). **a** UMAP plots of DeepImpute, DCA, MAGIC, SAVER, and raw data (scImpute, DrImpute, and VIPER) failed to run due to the large cell size of 48,267 cells). Colors represent original cell type labels as annotated. **b** Accuracy measurements of clustering using various metrics: adjusted Rand index (adjusted_rand_score), adjusted mutual information (adjusted_mutual_info_score), Fowlkes–Mallows Index (Fowlkes-Mallows), and Silhouette coefficient (Silhouette score). Higher values indicate better clustering accuracy. Bar colors represent different methods: DeepImpute (blue), DCA (orange), MAGIC (green), SAVER (red), and raw data (brown)

Another way to assess possible benefits of imputation is to conduct downstream functional analysis. Towards this, we utilized additional experimental and simulation datasets. We use an experimental dataset (Hrvatin) from GSE102827, composed of 48,267 annotated primary visual cortex cells from mice and which had 33 prior cell type labels [37]. Using the Seurat pipeline implemented in Scanpy, we extracted the UMAP [38] components (Fig. 4a). We then performed cell clustering using the Leiden clustering algorithm [39], an improved version of the Louvain algorithm [40]. We measure the accuracy of clustering assignments using various metrics, including the Adjusted Rand Index (ARI), the Adjusted Mutual Score (AMS), the Fowlkes-Mallow Index (FMI), and Silhouette Index (SI) to exam UMAP cluster shapes (Fig. 4b). Due to the size of the Hrvatin dataset, we could not run DrImpute and VIPER (speed issues) as well as scImpute (speed and memory issues), but only DeepImpute, DCA, MAGIC, and SAVER. DeepImpute manages to disentangle many clusters (Fig. 4a), resulting in the most improved clustering metrics compared to the scenario without imputation (Fig. 4b). DCA, the other deep neural-network-based method, also slightly improves the clustering metrics (Fig. 4b). On the contrary, MAGIC and SAVER decrease, rather than improving the clustering outcome. Notable, MAGIC manages to split many cell types but also highly distorts the data (Fig. 4a). SAVER disentangles some clusters, but also splits some clusters beyond the original cell type labels (Fig. 4a).

**Fig. 5:**
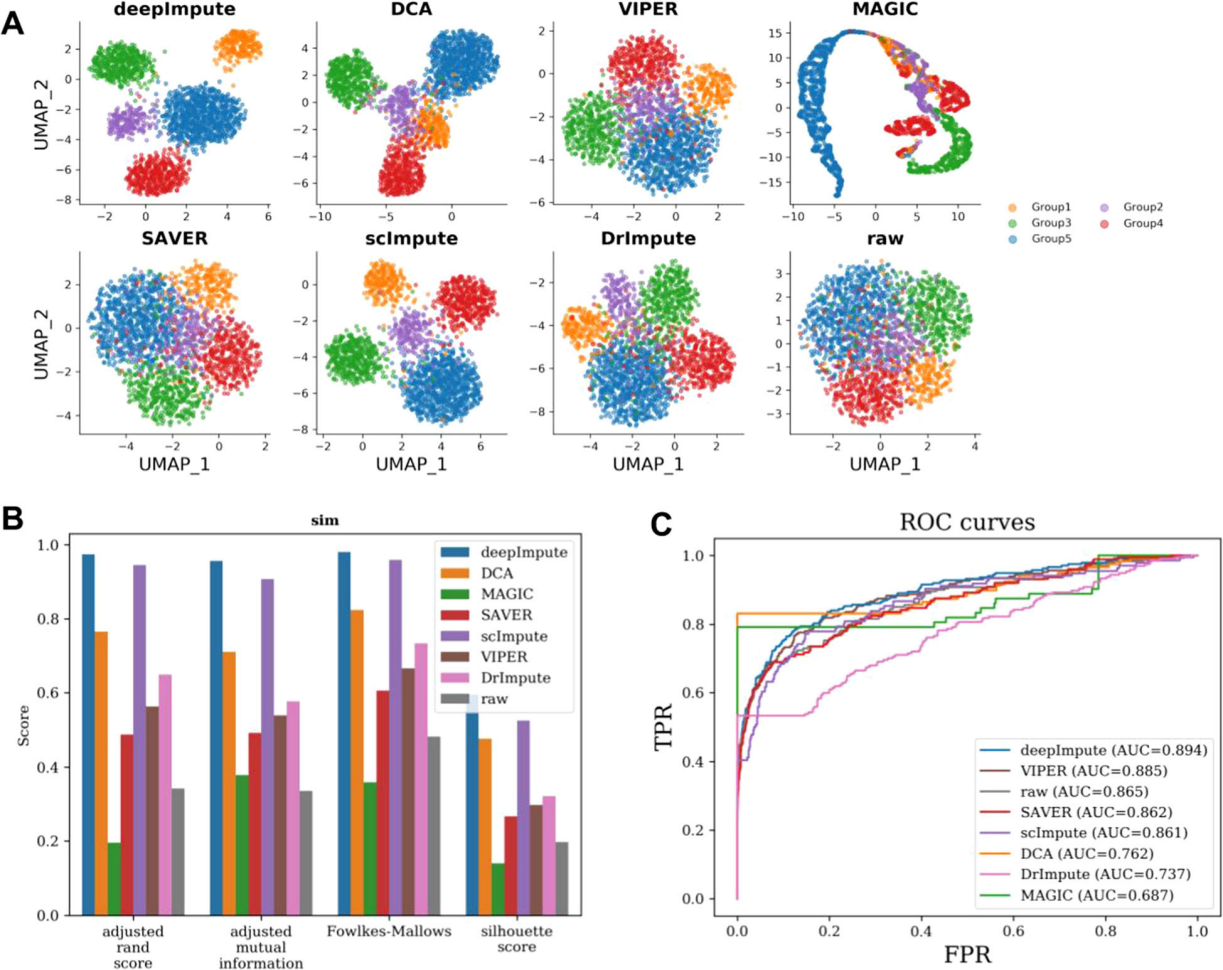
Comparison on effect of imputation on downstream function analysis of simulated data using Splatter. This simulation dataset is composed of 4000 genes and 2000 cells, split into 5 cell types (proportions: 5%/5%/10%/20%/20%/40%). **a** UMAP plots of DeepImpute, MAGIC, SAVER, scImpute, DrImpute, and raw data. Each color represents one of the 5 cell types. **b** Accuracy measurements of clustering using the same metrics as in Fig. 4b. Bar colors represent different methods as shown in the figure. **c** Accuracy measurements of differentially expressed genes by different imputation methods. The top 500 differentially expressed genes in each cell type are used to compare with the true differentially expressed genes in the simulated data, over a range of adjusted *p* values for each method. Colors represent different methods as shown in the figure

Given lack of absolute truth of class labels in experimental data, we next generated a simulation data using Splatter. This simulation dataset (sim) is composed of 4000 genes and 2000 cells, which are split into 5 cell types (proportions: 5%/5%/10%/20%/20%/40%). DeepImpute successfully separates cell types on the simulation, closely followed by scImpute (Fig. 5a). These observations are confirmed by the evaluation metrics, where DeepImpute achieves almost perfect scores for ARI, AMS, and FMI and significantly increases the Silhouette score compared to the raw data (Fig. 5b). Next, we compare all seven imputation methods for their capabilities to recover differentially expressed genes in the simulation data (Fig. 5c). For each method, we extracted the top 500 differentially expressed genes in each cell type and compared with the true differentially expressed genes. Overall, DeepImpute has the highest precision (AUC = 0.893) at detecting differentially expressed genes, compared to those of no imputation and other imputation methods. All together, these results from both experimental and simulation data show unanimously that DeepImpute improves downstream functional analysis.

### DeepImpute is a fast and memory efficient package

As scRNA-seq becomes more popular and the number of sequenced cells scales exponentially, imputation methods will have to be computationally efficient to be widely adopted. With such a goal in mind, we choose the Mouse1M dataset to evaluate the computational speed and memory usage among different imputation methods. We use Mouse1M dataset as it has the highest number of cells to assess how adaptive each method is.

**Fig. 6:**
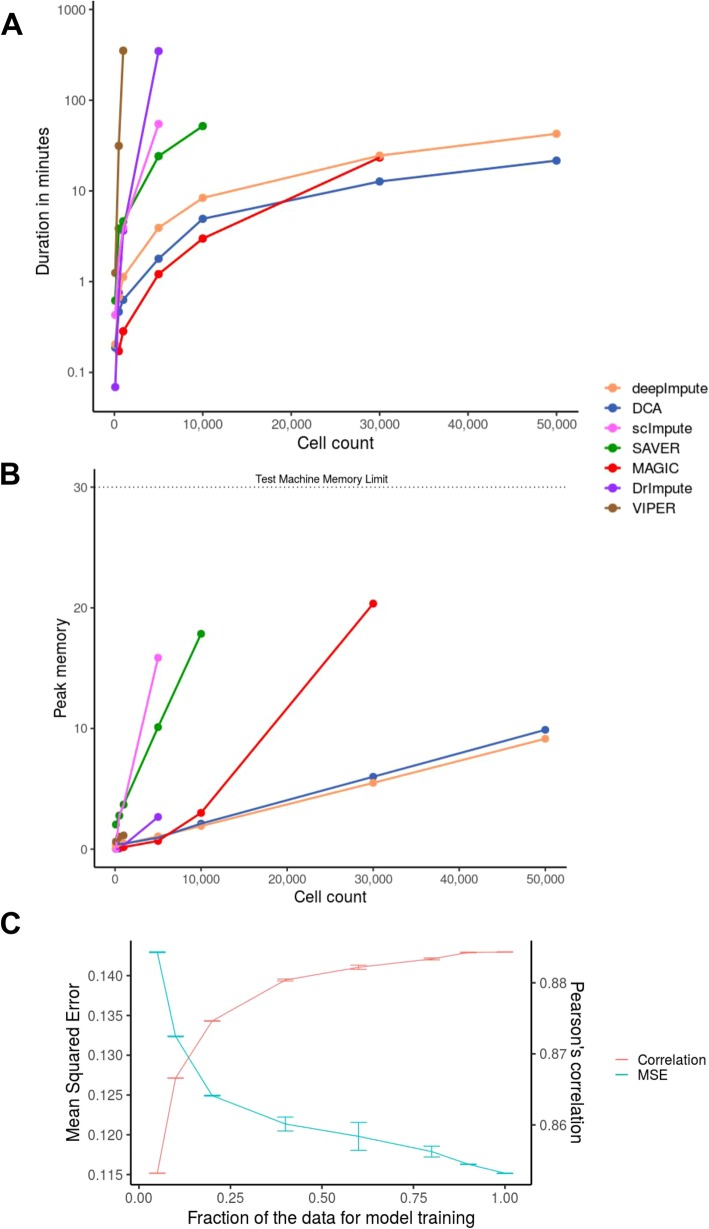
Speed and memory usage comparison among imputation methods, as well as the effect of subsampling training data on DeepImpute accuracy. **a**, **b** Speed and memory comparisons on the Mouse1M dataset. This dataset is chosen for its largest cell numbers. Color labels different imputation methods. **a** Speed average over 3 runs. The *x*-axis is the number of cells, and the *y*-axis is the running time in minutes (log scale) of the imputation process. **b** RAM memory usage. The *x*-axis is the number of cells, and the *y*-axis is the maximum RAM used by the imputation process. Because of the limited amount of memory or time, scImpute, SAVER, and MAGIC exceeded the memory limit respectively at 10k, 30k, and 50k cells, thus no measurements at these and higher cell counts. VIPER and DrImpute each exceeded 24 h on 1k and 10k cells; therefore, they too do not have measurements at these and higher cell counts. **c** The effect of subsampling training data on DeepImpute accuracy. Neuron9k dataset is masked and measured for performance as in Fig. 2. *x*-axis is the fraction of cells in the training data set, and *y*-axis labels are values for mean squared error (left) and Pearson’s correlation coefficient (right). Color labels are as indicted in the graph. Error bars represent the standard deviations over the 10 repetitions

We downsampled the Mouse1M data, ranging in size from 100 to 50k cells (100, 500, 1k, 5k, 10k, 30k, 50k). We ran the imputations three times and measured the runtime (for both training and testing steps) and memory load on an 8-core machine with 30 GB of memory. DeepImpute, DCA, and MAGIC outperformed the other four packages on speed (Fig. 6a), and DeepImpute is the most advantageous when the cell counts get large (> 30k). DCA is consistently and slightly slower than DeepImpute through all tests. The other four imputation methods (scImpute, DrImpute, VIPER, and SAVER) are significantly slower and consume significantly more memory (Fig. 6b). The slow computation time of VIPER and DrImpute are due to lack of parallelization. VIPER is unable to scale beyond 5k cells within 24 h, while scImpute exceeded the 30 GB of memory available and failed to run on more than 10k cells. For memory, DeepImpute and DCA, two neural-network-based methods, are the most efficient, and their merits are much more pronounced on large datasets (Fig. 6b). MAGIC uses a similar amount of memory as DeepImpute and DCA on smaller datasets; however, as the dataset size increases beyond 10k cells, it requires significantly more memory. It hits an out of memory error and is unable to finish the 50k cell imputation on our 30GB machine. In all, judging by both computation speed and memory efficiency on larger datasets, DeepImpute and DCA tops the other five methods.

### DeepImpute is a scalable machine learning method

Unlike the other imputation methods, DeepImpute first fits a predictive model and then performs imputation separately. The model fitting step uses most of the computational resources and time, while the prediction step is very fast. We then asked the question what is the minimal fraction of the dataset needed to train DeepImpute and obtain efficient imputation without extensive training time. Hence, we used the neuron9k dataset and evaluated the effect of different subsampling fraction (5%, 10%, 20%, 40%, 60%, 80%, 90%, 100%) in the training phase on the imputation prediction phase. We randomly picked a subset of the samples for the training step and computed the accuracy metrics (MSE, Pearson’s correlation coefficient) on the whole dataset, with 10 repetitions under each condition. Model performance improvement begins to slow down at around 40% of the cells (Fig. 6c). Specifically, from 40 to 100% fraction of data as the training set, the MSE decreases slightly from 0.121 to 0.116, and Pearson’s coefficient score marginally improves from 0.880 to 0.884. These experiments demonstrate another advantage of DeepImpute over the other competing methods, that is, the use of only a fraction of the data set will reduce the running time even more with little sacrifice to the accuracy of the imputed results.

## Discussion

Dropout values in scRNA-seq experiments represent a serious issue for bioinformatic analyses, as most bioinformatics tools have difficulty handling sparse matrices. In this study, we present DeepImpute, a new algorithm that uses deep neural networks to impute dropout values in scRNA-seq data. We show that DeepImpute not only has the highest overall accuracy using various metrics and a wide range of validation approaches, but also offers faster computation time with less demand on the computer memory. In both simulated and experimental datasets, DeepImpute shows benefits in increasing clustering results and identifying significantly differentially expressed genes, even when other imputation methods are not desirable. Furthermore, it is a very “resilient” method. The model trained on a fraction of the input data can still yield decent predictions, which can further reduce the running time. Together, these results demonstrate consistently and robustly that DeepImpute is an accurate and highly efficient method, and it is likely to withstand the tests of time, given the rapid growth of scRNA-Seq data volume.

Through systematic comparisons, two deep-learning-based methods, DeepImpute and DCA, show overall advantages over other methods, between which DeepImpute performs even better. Several unique properties of DeepImpute contribute to its superior performance. One of them is using a divide-and-conquer approach. This approach has several benefits. First, contrary to an auto-encoder as implemented in DCA, the subnetworks are trained without using the target genes as the input. It reduces overfitting while enforcing the network to understand true relationships between genes. Second, splitting the genes into subsets results in a lower complexity in each sub-model and stabilizing neural networks. As a result, a small change in the hyperparameters has little effect on the result. Using a single set of hyperparameters, DeepImpute achieves the highest accuracies in all four experimental datasets (Fig. 2a). Third, splitting the training into sub-networks results in increased speed as there are fewer input variables in each subnetwork. Also, training of each sub-network is done in parallel on different threads, which is more difficult to do with one single neural network.

Unlike some other imputation algorithms in comparison, DeepImpute is a machine learning method. The training and the prediction processes of DeepImpute are separate, and this may provide more flexibility when handling large datasets. Moreover, we have shown that using only a fraction of the overall samples, one can still obtain decent imputation results without sacrificing the accuracy of the model much, thus further reducing the running time. Perhaps, another advantage of DeepImpute over other methods is that one can pre-train a dataset of a cell type (or cell state) on another cell type (or cell state) decently. This pre-training process is very valuable in some cases, such as when the number of cells in the dataset is too small to construct a high-quality model. Pre-training can also largely reduce the overall computation time, since DeepImpute spends most of the time on training the samples. Thus, it is also a good strategy when the new, large dataset is very similar to the dataset used in pre-training.

An enduring imputation method has to adapt to the ever-increasing volume of scRNA-seq data. DeepImpute is such a method, implemented in deep learning framework where new solutions for speed improvements keep appearing. One example is the development of neural network-specific hardware (such as tensor processing units [41], or TPUs) which are now available on Google Cloud. TPU can dramatically accelerate the tensor operations and thus the imputation process. We were already able to deploy DeepImpute in a Google Cloud environment where TPUs are already available. Another example is the development of frameworks that efficiently use computer clusters to parallelize tasks such as Apache-Spark [42] or Dask [43]. Such resources will help DeepImpute and similar deep-learning methods, such as scDeepCluster designed for clustering analysis [44], achieve even higher peed over time and keep up with the development of scRNA-seq technologies.

## Methods

### The workflow of DeepImpute

DeepImpute is a deep neural network-based imputation workflow, implemented with the Keras [45] framework and TensorFlow [46] in the backend. Below, we describe the workflow in four steps: preprocessing, architecture, training procedure, and imputation.

#### Preprocessing

The first step of DeepImpute is selecting the genes for imputation, based on the variance over mean ratio (default = 0.5), which are deemed interesting for downstream analyses [47, 48]. For efficiency, we adopt a divide-and-conquer strategy in our deep learning imputation process. We split the genes into *N* random subsets, each with *S* numbers of genes, which we call “target genes.” By default, *S* is set as 512. If the number of target genes is not a multiple of this number, we round the number of genes to impute in *N* + 1 subsets of deep neural networks. The details of this step are illustrated in Additional file 1: Figure S1.

#### Network architecture

For each subset, we train a neural network of four layers: the input layer of genes that are correlated to the target genes, a 256-neuron fully connected hidden layer with a rectified linear unit (ReLU) activation function, a *dropout* layer (note: different from dropout data in scRNA-Seq), and an output layer of *S* target genes. A gene is selected to the input layer, if it satisfies these conditions: (1) it is not one of the target genes and (2) it has top 5 ranked Pearson’s correlation coefficient with a target gene. The *dropout* layer is included after the hidden layer, as a common strategy to prevent overfitting [49]. We optimized the *dropout* rate as 20%, after experimenting the *dropout* rates from 0 to 90% (Additional file 2: Figure S1). The other default parameters of the networks include a learning rate of 0.0001, a batch size of 64, and a subset size of 512. As internal controls, we also experimented two alternative setups for DeepImpute: one with the same architecture but linear activation function and the other one without hidden layers of neurons.

#### Training procedure

The training starts by splitting the cells between a training (95%) and a test set (5%). The test set is used at each epoch to measure overfitting. We use a weighted mean squared error (MSE) loss function that gives higher weights to genes with higher expression values. This emphasizes accuracy on high confidence values and avoids over penalizing genes with extremely low values (e.g., zeros). For a given cell c, the loss is calculated as follows:

$$ \mathrm{Loss}\_\mathrm{c}\kern0.5em =\sum \kern0.5em {Y}_i\cdotp {\left({Y}_i-{\hat{Y}}_i\right)}^2 $$

where *Y*_*i*_ is the value of gene *i* for cell c and ${\hat{Y}}_i$ is the corresponding estimated value at a given epoch. For the gradient descent algorithm, we choose an adaptive learning rate method, the Adam optimizer [50], since it is known to perform very efficiently over sparse data [51]. The training stops if it reaches 500 epochs or if the training does not improve for 10 epochs.

#### Imputation

Once the network weights are properly trained, we impute the data by filling zeros in the original matrix with the imputed values.

### Evaluation metrics

#### Accuracy comparison on real datasets

To evaluate the accuracy of imputation, we apply a random mask to the real single-cell datasets. The masking probability function is estimated in a similar fashion as in splatter [14]. For each gene, we extract the proportion of zeros vs. the mean of those positive values. As done in Splatter, we fit a logistic function to these data points. Next, for each gene in the dataset, we mask ten cells at random using a multinomial distribution: each cell *c*_*1*_*... c*_*n*_ has a dropout probability *p*_*1*_*... p*_*n*_ given by the logistic function previously fitted. The masked cells are sampled from a multinomial distribution with parameters (*q*_*1*_, *q*_*2*_*,..., q*_*n*_), where *q*_*i*_ = *p*_*i*_/∑_*i*_*p*_*i*_  are the normalized probability such that ∑_*i*_*q*_*i*_ = 1.

These original values are used as “truth values” to evaluate the performance of imputation methods. We used two types of performance metrics: the overall Pearson correlation coefficient and MSE, both on log transformed counts. When needed, we also computed MSE between cells *c*_*j*_ and between genes *g*_*i*_. *Speed and memory comparison:* we run comparisons on a dedicated 8-core, 30-GB RAM, 100-GB HDD, Intel Skylake machine running Debian 9.4. We record process memory usage at 60-s intervals. For testing data, we use the Mouse1M dataset since it has the largest number of single cells (Additional file 3: Table S1). We filter out genes that are expressed in less than 20% of cells, leaving 3205 genes in our sample. From this dataset, we generate 7 subsets ranging in size (100, 500, 1k, 5k, 10k, 30k, 50k cells). We run each package 3 times per subset to estimate the average computation time. Some packages (VIPER, DrImpute, SAVER, scImpute, and MAGIC) are not able to successfully handle the larger files either due to out-of-memory errors (OOM) or exceedingly long run times (> 24 h).

### Downstream functional analysis

#### Clustering

We perform cell clustering using the Seurat pipeline implemented in Scanpy. After preprocessing the data, we extract the UMAP components [38] and cluster the cells using the Leiden algorithm recommended in the Scanpy documentation. To assess the performance of the clusters, we use four metrics. For all of them, a value of 1 indicates a perfect clustering, while 0 corresponds to random assignments.

#### Adjusted mutual information [16]

It is an entropy-based metric that calculates the shared entropy between two clustering assignments, and is adjusted for chance. The mutual information is calculated by $MI\left(C,K\right)={\sum}_{i\in C}{\sum}_{j\in K}P\left(i,j\right)\cdotp \mathit{\log}\left(\frac{P\left(i,j\right)}{P(i)P(j)}\right)$, where *P*(*i*, *j*) is the probability of cell *i* belonging to both cluster C and K.

#### Adjusted Rand index [16]

It is the ratio of all cell pairs that are either correctly assigned together or correctly not assigned together, among all possible pairs. It is also adjusted for chance.

#### Fowlkes–Mallows index

It is a metric derived from the true positives (TP), false positives (FP), and false negatives (FN) as follows: $\mathrm{FMI}=\sqrt{\frac{\mathrm{TP}}{\mathrm{TP}+\mathrm{FP}}\cdotp \frac{\mathrm{TP}}{\mathrm{TP}+\mathrm{FN}.}}$

#### Silhouette coefficient [29]

It is a clustering metric derived by comparing the mean intra-cluster distance and the mean inter-cluster distance.

#### Differential expression analysis

We perform the differential expression analysis using the scanpy package on the simulation as the groups are pre-defined. For each method, we extracted the differentially expressed genes for each cell group by performing a *t* test of one group against the rest groups. We used Benjamini-Hochberg correction for multiple hypothesis testing to obtain adjusted *p* value (pval_adj_). Since each method has generated different differentially expressed genes, we extracted the top 500 differentially expressed genes for each group and pooled the differentially expressed genes for all of the groups. Using 1-pval_adj_ as the DE calling probability and the true differentially expressed genes (by Splatter) as the truth measure, we calculated the area under the curve (AUC) for the ROC curve for each method using the scikit-learn python package.

### RNA FISH validation

We obtain a Drop-Seq dataset (GSE99330) and its RNA FISH dataset from a melanoma cell line, as described by Torre et al. [36]. The summary of the dataset is listed in Additional file 3: Table S1. For the comparison between RNA FISH and the corresponding Drop-Seq experiment, we keep genes with a variance over mean ratio > 0.5, the same as other datasets in this study, leaving six genes in common between the FISH and the Drop-Seq datasets.

For GINI coefficient calculation, we first normalize the cells in each dataset using a housekeeping gene (glyceraldehyde 3-phosphate dehydrogenase, or GAPDH)-based factor, as done by others [20]. We remove GAPDH outlier cells (defined here as the cells below the 10th and above the 90th percentiles). Then, we rescale each data point by a GAPDH-based factor, as follows:

$$ \mathrm{data}\left[\mathrm{cell},\mathrm{gene}\right]=\mathrm{data}\left[\mathrm{cell},\mathrm{gene}\right]\ast \mathrm{factor}\left(\mathrm{cell}\right) $$

$$ \mathrm{where}\ \mathrm{factor}\left(\mathrm{cell}\right)=\mathrm{mean}\left(\mathrm{data}\left[:,\mathrm{GAPDH}\right]\right)/\mathrm{data}\left[\mathrm{cell},\mathrm{GAPDH}\right] $$

Then, we compute GINI coefficient, as done in SAVER [20]. For distribution normalization, the procedure is the same except that we first normalize each gene by an efficiency factor (defined as the ratio between its mean value for FISH and its value for the imputation method). We calculate the MSEs and Pearson’s coefficients with the following formulas:

$$ \mathrm{MSE}\left(\mathrm{gene},\mathrm{method}\right)={\sum}_{\mathrm{cell}}{\left(\ {X}_{\mathrm{FISH}}\left(\mathrm{gene},\mathrm{cell}\right)-{X}_{\mathrm{method}}\left(\mathrm{gene},\mathrm{cell}\right)\ \right)}^2 $$

$$ \mathrm{Corr}\left(\mathrm{gene},\mathrm{method}\right)=\frac{\mathrm{Cov}\left(\ {X}_{\mathrm{FISH}}\left(\mathrm{gene}\right),{X}_{\mathrm{method}}\left(\mathrm{gene}\right)\ \right)}{\mathrm{Var}\left(\ {X}_{\mathrm{FISH}}\left(\mathrm{gene}\right)\ \right)\cdotp \mathrm{Var}\left(\ {X}_{\mathrm{method}}\left(\mathrm{gene}\right)\ \right)} $$

where *X* is the input matrix of gene expression from RNA-FISH or Drop-Seq, Cov is the covariance, and Var is the variance.

## Supplementary information

**Additional file 1.** Explanatory figures for DeepImpute’s preprocessing and for the masking experiment.

**Additional file 2.** Dropout and activation function optimization experiments for DeepImpute’s architecture.

**Additional file 3.** Summary table of the dataset used in this paper.

## Data Availability

*scRNA-seq Datasets* In this study, we evaluate imputation metrics on nine datasets. Four of them (Jurkat, 293T, neuron9k, and Mouse1M) are downloaded from the 10X Genomics support website (https://support.10xgenomics.com/single-cell-gene-expression/datasets). Briefly, the Jurkat dataset is extracted from the Jurkat cell line (human blood). 293T is a blood cell line derived from HEK293T that expresses a mutant version of the SV40 large T antigen. The neuron9k dataset contains brain cells from an E18 mouse. Mouse1M also contains brain cells from an E18 mouse. The FISH and GSE99330 data were both extracted from the same melanoma cell line WM989-A6 [36]. Two other datasets are taken from GSE67602 [52], composed of mouse interfollicular epidermis cells and the Hrvatin dataset GSE102827 [37] dataset, extracted from primary visual cortex of C57BL6/J mice. Additionally, we simulate a dataset with the Splatter package [14] with parameters dropout.shape = − 0.5, dropout.mid = 1, 4000 genes and 2000 cells split into 5 groups with proportions 10%, 10%, 20%, 20%, and 40%. Each gene in each group is automatically assigned a differential expression (DE) factor, where 1 is not differentially expressed, a value less than 1 is downregulated, and more than 1 is upregulated. *Third party software* For comparison, we use the latest version of SAVER (v1.1.1) at https://github.com/mohuangx/SAVER/releases, ScImpute (v0.0.9) at https://github.com/Vivianstats/scImpute, DrImpute (v1.0) available as a CRAN package, MAGIC (v1.4.0) at https://github.com/KrishnaswamyLab/magic, VIPER (v1.0) at https://github.com/ChenMengjie/VIPER/releases, and DCA (0.2.2) at https://github.com/theislab/dca. We preprocess the datasets according to each method’s standard: using a square root transformation for MAGIC, log transformation for DeepImpute (with a pseudo count of 1), but raw counts for scImpute, DrImpute, SAVER, and DCA. For VIPER, we remove all genes with a null total count and rescale each cell to a library size of one million (RPM normalization) as recommended. *DeepImpute’s material* DeepImpute package and its documentation are freely available on GitHubhttps://github.com/lanagarmire/DeepImpute [53] under the MIT license. The software as well as the source code to reproduce the figures of this paper was deposited on Zenodo 10.5281/zenodo.3459902 [54].
