# Supplementary material for: DeepImpute: an accurate, fast, and scalable deep neural network method to impute single-cell RNA-seq data
Source: Genome Biol. 2019 Oct 18;20:211. doi: 10.1186/s13059-019-1837-6 (PMC6798445; doi:10.1186/s13059-019-1837-6)
Supplement: Supplementary file 1 — Additional file 1. Explanatory figures for DeepImpute’s preprocessing and for the masking experiment. [file 13059_2019_1837_MOESM1_ESM.docx]

Additional file 1


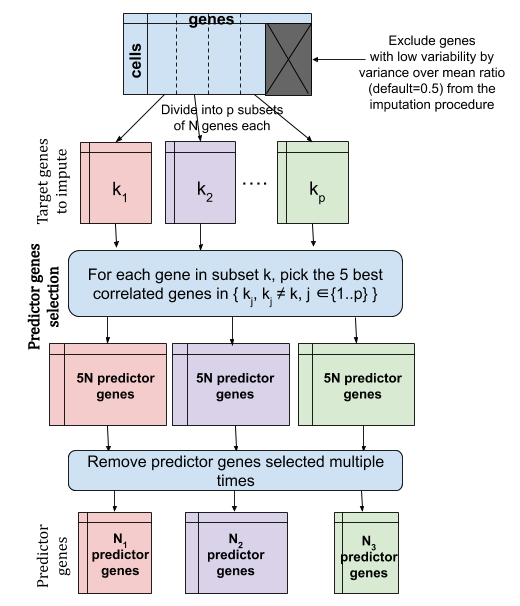


**Figure S1:** Preprocessing steps for DeepImpute. DeepImpute starts by selecting “target genes” that meet a variability criteria based on variance over mean ratio (default =0.5), and then splits them into subsets with the same number of genes (default: N = 512). For the last subset of genes less than the default value, they are rounded into the next sub-neural network model. These target genes make up the output layer of the sub-neural network, whose zero values are imputed. For each each target gene g_i_ in subset k, we select the 5 best correlated genes, or predictor genes, which are not part of the target genes in the subset k. Finally, we remove those predictor genes that were selected multiple times in all the sub-neural networks. We use the remaining predictor genes as the input layer for the sub-neural network.


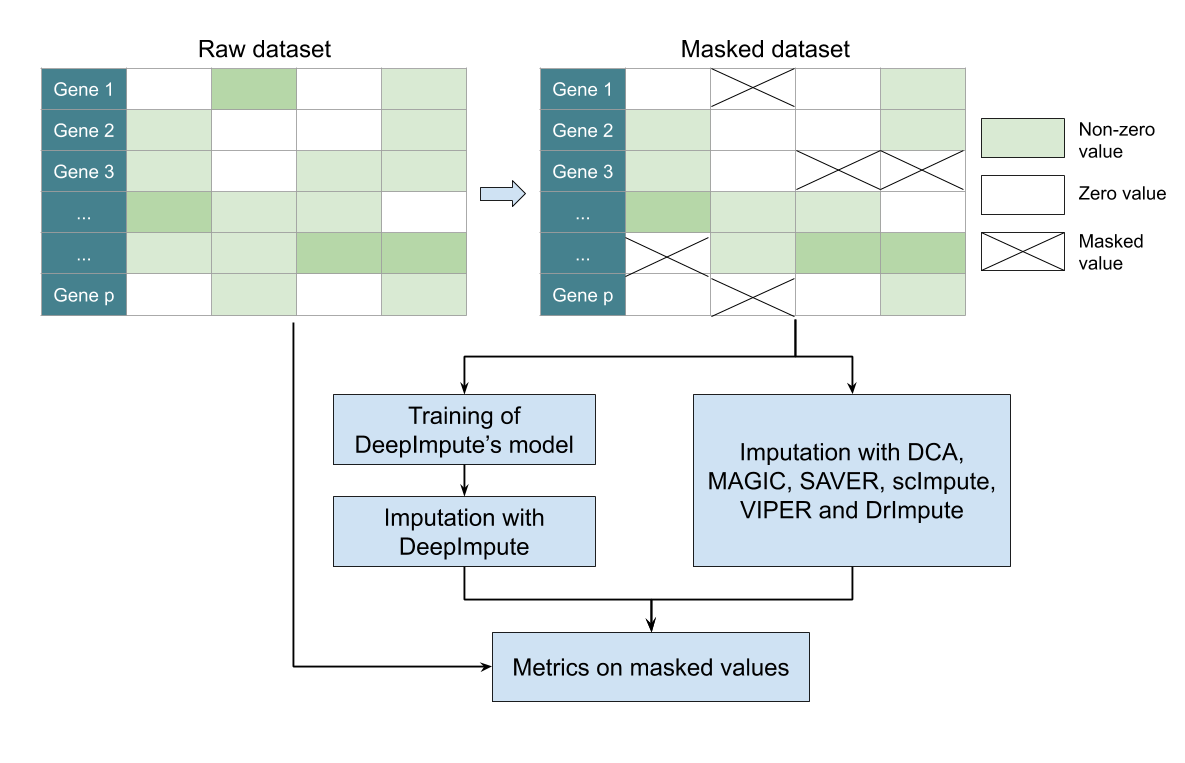


**Figure S2:** The illustration of masking in single cell RNA-Seq data.
