# Supplementary material for: DeepImpute: an accurate, fast, and scalable deep neural network method to impute single-cell RNA-seq data
Source: Genome Biol. 2019 Oct 18;20:211. doi: 10.1186/s13059-019-1837-6 (PMC6798445; doi:10.1186/s13059-019-1837-6)
Supplement: Supplementary file 2 — Additional file 2. Dropout and activation function optimization experiments for DeepImpute’s architecture. [file 13059_2019_1837_MOESM2_ESM.docx]

Additional file 2


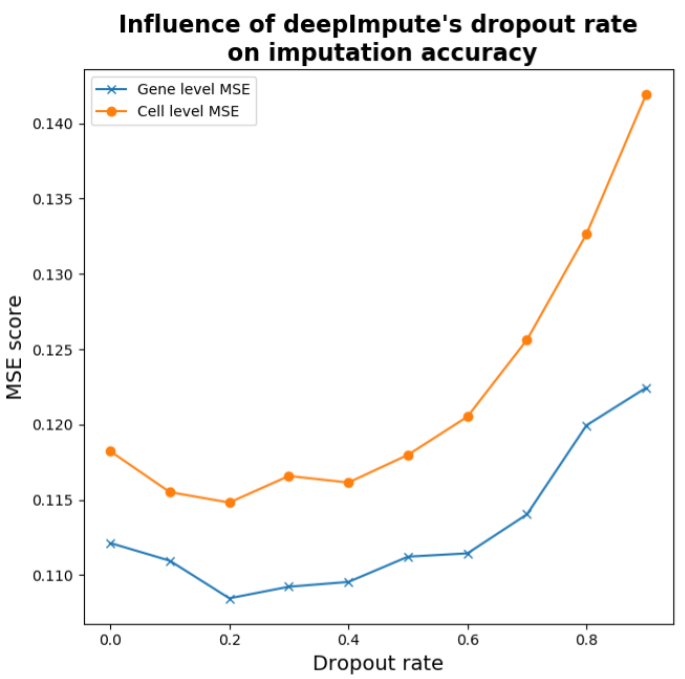


**Figure S1:** The effect of dropout rate in the hidden layer of auto-encoder on imputation. The MSE scores for dropout rates varying from 0 to 90% are shown. The blue and orange lines are gene-level and cell-level MSEs, respectively.


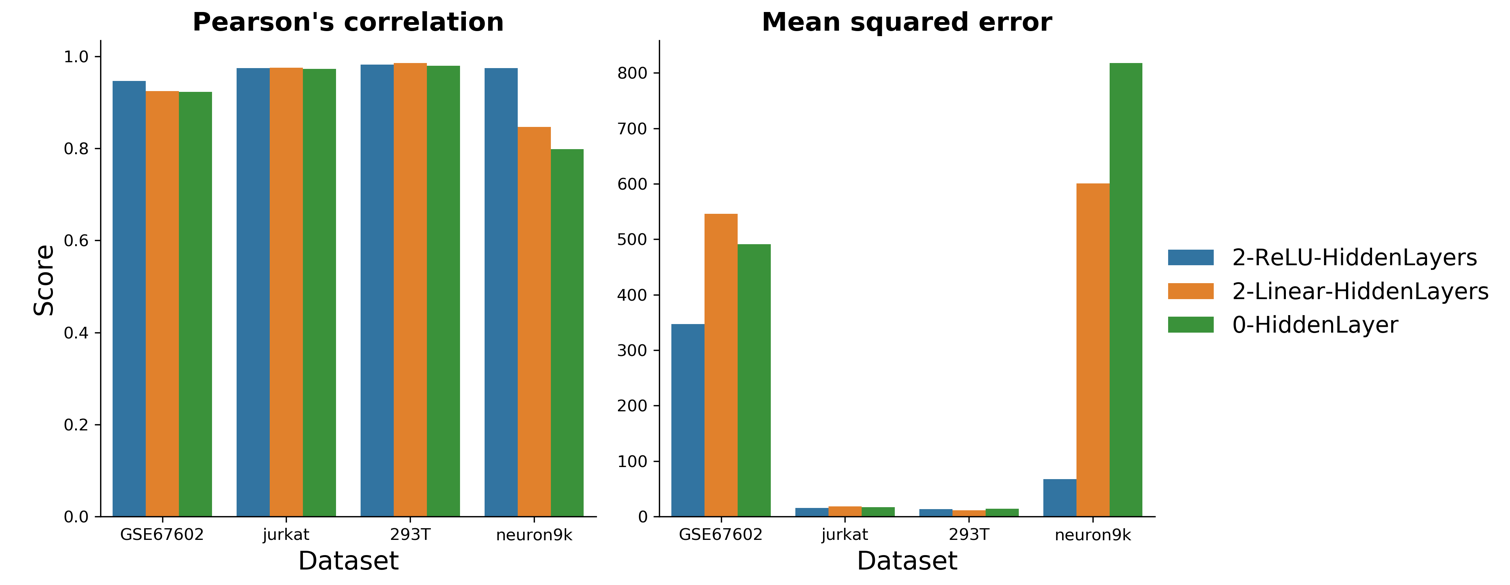


**Figure S2:** Accuracy comparison between DeepImpute and two other variant architectures. Bar plots of Pearson’s correlation coefficients (left) and mean squared error (right) are shown for the masked data points of each dataset in Figure 2. Three DeepImpute variants are compared: default DeepImpute with ReLU activation function (blue), DeepImpute with linear activation function (orange), and DeepImpute without hidden layers (green).
