# Supplementary material for: DeepImpute: an accurate, fast, and scalable deep neural network method to impute single-cell RNA-seq data
Source: Genome Biol. 2019 Oct 18;20:211. doi: 10.1186/s13059-019-1837-6 (PMC6798445; doi:10.1186/s13059-019-1837-6)
Supplement: Supplementary file 3 — Additional file 3. Summary table of the dataset used in this paper. [file 13059_2019_1837_MOESM3_ESM.docx]

Additional file 2

| **Dataset** | **# Cells** | **Sample type** | **Organism** | **Accession** |
| --- | --- | --- | --- | --- |
| Jurkat | 3,258 | Blood cell line | Homo Sapiens | 10X Genomics* |
| 293T | 2,885 | Blood cell line | Homo Sapiens | 10X Genomics* |
| Neuron9k | 9,128 | Brain cells | Mus Musculus | 10X Genomics* |
| GSE67602 | 1,422 | Interfollicular  Epidermis cells | Mus Musculus | GEO (GSE67602) |
| Mouse1M | 1,306,127 | Brain cells | Mus Musculus | 10X Genomics* |
| FISH | 88,040 | Melanoma cell line | Homo Sapiens | Torre et al. |
| GSE99330 | 8,641 | Melanoma cell line | Homo Sapiens | GEO (GSE99330) |
| Sim | 2,000 | N/A | N/A | N/A |
| Hrvatin | 48,267 | Primary visual cortex | Mus Musculus | GEO (GSE102827) |

**Table S1:** Summary of the single-cell datasets

*: the URL to access the dataset is:

<https://support.10xgenomics.com/single-cell-gene-expression/datasets>
